# Multi-omics characterization and validation of invasiveness-related molecular features across multiple cancer types

**DOI:** 10.1186/s12967-021-02773-x

**Published:** 2021-03-25

**Authors:** Guoshu Bi, Jiaqi Liang, Yuansheng Zheng, Runmei Li, Mengnan Zhao, Yiwei Huang, Cheng Zhan, Songtao Xu, Hong Fan

**Affiliations:** 1 Department of Thoracic Surgery, Zhongshan Hospital, Fudan University, No. 180 Fenglin Rd, Xuhui District, Shanghai, 200032 China; 2 Department of Biostatistics, Public Health, Fudan University, Shanghai, 200000 China

**Keywords:** Invasiveness, Pan-cancer, Multi-omics, Molecular features, Immunity

## Abstract

**Background:**

Tumor invasiveness reflects many biological changes associated with tumorigenesis, progression, metastasis, and drug resistance. Therefore, we performed a systematic assessment of invasiveness-related molecular features across multiple human cancers.

**Materials and methods:**

Multi-omics data, including gene expression, miRNA, DNA methylation, and somatic mutation, in approximately 10,000 patients across 30 cancer types from The Cancer Genome Atlas, Gene Expression Omnibus, PRECOG, and our institution were enrolled in this study.

**Results:**

Based on a robust gene signature, we established an invasiveness score and found that the score was significantly associated with worse prognosis in almost all cancers. Then, we identified common invasiveness-associated dysregulated molecular features between high- and low-invasiveness score group across multiple cancers, as well as investigated their mutual interfering relationships thus determining whether the dysregulation of invasiveness-related genes was caused by abnormal promoter methylation or miRNA expression. We also analyzed the correlations between the drug sensitivity data from cancer cell lines and the expression level of 685 invasiveness-related genes differentially expressed in at least ten cancer types. An integrated analysis of the correlations among invasiveness-related genetic features and drug response were conducted in esophageal carcinoma patients to outline the complicated regulatory mechanism of tumor invasiveness status in multiple dimensions. Moreover, functional enrichment suggests the invasiveness score might serve as a predictive biomarker for cancer patients receiving immunotherapy.

**Conclusion:**

Our pan-cancer study provides a comprehensive atlas of tumor invasiveness and may guide more precise therapeutic strategies for tumor patients.

**Supplementary Information:**

The online version contains supplementary material available at 10.1186/s12967-021-02773-x

## Background

For decades, cancer has been one of the leading causes of deaths worldwide [1], while the activating invasion is one of the six hallmark capabilities of the cancer [2] and greatly worse the patients’ prognosis. However, the biological mechanisms underlying invasiveness and metastasis were largely an enigma. An in-depth exploration of the tumor invasiveness-related mechanisms would be of great significance for us to understand tumorigenesis process and identify potential therapeutic targets, thus improving cancer patient survival.

Different tumors have different invasiveness-associated molecular mechanisms but share many common genes and signaling pathways [3]. Research in this area has accelerated dramatically recently as powerful new tools and refined experimental models have become available. In recent years, pan-cancer analysis projects of specific function and biological pathway genes aimed at identifying the common molecular features across multiple tumor types have been increasingly reported and remarkably provided a multi-dimensional, in-depth, and comprehensive understanding of human cancer [4–8]. For example, the multi-omics study of Ye et al. identified common molecular alterations associated with tumor hypoxia and analyzed their correlation with sensitivity to a series of anti-cancer drugs [4], thus pointing out the direction of research into tumor hypoxia microenvironment in the post-genome era and highlighting the need to take tumor hypoxia status into consideration in future studies. Luo et al. focused on telomerase reverse transcriptase activation in 8 cancer types and developed a random forest classifier integrating multi-omics signatures to identify patients with different telomerase activities and overall survival rates, providing novel insights that link telomerase-related signatures to patient survival and opening new avenues for treating cancer [5]. Although several invasiveness-associated gene signatures have been reported, a comprehensive investigation of invasiveness-related molecular features across multiple cancer types has not yet been explored.

In the present study, we provide an in-depth pan-cancer analysis of invasiveness-associated dysregulated molecular features and describe their intersection and mutual regulatory mechanisms based on the genomic, epigenomic, transcriptomic, proteomics, metabolites, and drug-response profiles of 30 cancer types from available databases and our institution. An invasiveness score based on a 24-gene signature was established and validated to serve as a robust prognostic factor and predictive biomarker for guiding more precise and personalized anti-cancer therapeutic strategies.

## Methods and materials

### Acquisition of pan-cancer multi-omics datasets

Level 4 gene sequencing (FPKM normalized), mature microRNA (miRNA) expression, protein expression, DNA methylation, somatic mutation, copy number variation (CNV), and corresponding clinical data of 33 human cancers in The Cancer Genome Atlas (TCGA) were downloaded from the UCSC Xena browser (GDC hub: https://gdc.xenahubs.net). We removed patients whose clinical outcome information including survival time and vital status were vague or absent. We also downloaded mass proteomic spectrum data from the Clinical Proteomic Tumor Analysis Consortium (CTPAC) [9, 10]. Normalized metabolite levels of several TCGA breast cancer (BRCA) samples were obtained from the study by Tang et al. [11]. We also downloaded cancer cell line drug sensitivity databases from the Genomics of Drug Sensitivity in Cancer (GDSC; available at https://www.cancerrxgene.org/downloads/anova) [12–14], which includes gene expression data obtained using an Affymetrix HT HG U133A array and drug sensitivity data, presented as area under the dose–response curve (AUC) values and IC_50_ values (half maximal inhibitory concentration) based on cell viability assays. Geeleher et al. developed a novel computational method to determine drug response in large clinical cancer genomics datasets, enabling us to conduct pharmacogenomics discovery in TCGA patients without having to collect their actual drug response information [15]. We combined the results from MiRWalk (http://mirwalk.umm.uni-heidelberg.de/search_mirnas/) and TargetScan (http://www.targetscan.org/) to identify potential targeting relationships between genes and miRNAs [16].

We also included several independent gene-array datasets from Gene Expression Omnibus (GEO) and PRECOG repository as external validation cohorts (Additional file 1: Table S1). Gene expression data and corresponding reliable clinical survival information were directly downloaded from http://www.ncbi.nlm.nih.gov/geo and https://precog.stanford.edu/, respectively. Background adjustments and data normalization were performed with the *limma* package [17].

We retrospectively selected 34 patients with lung adenocarcinoma (LUAD) and 44 with lung squamous cell carcinoma (LUSC) who underwent lobectomy and systematic lymph node resection at our institution, from July 2012 to December 2012. All pulmonary resections were performed by experienced thoracic surgeons in our institution, and resected tumors and lymph node specimens were all labeled in the operating theater and reviewed by at least two qualified pathologists to confirm the diagnosis of LUAD or LUSC through hematoxylin and eosin-stained sections and immunochemical analysis. Patients with evidence of metastasis at the time of diagnosis, or history of chemotherapy, radiotherapy, and immunological therapy were excluded. RNA sequencing for all tumor samples was performed using Illumina Hiseq 2500 and BGI-500RNAseq platforms. Patients’ postoperative data were collected annually by outpatient follow-up and phone call. All patients provided written informed consent to conduct genomic studies in accordance with the ethical principles of the Declaration of Helsinki and the International Conference on Harmonization Guidelines for Good Clinical Practice, and the study was approved by the ethical committees of our institution (No. 201986122 and No. 2011-219(2)).

### Estimation of invasiveness-score

To construct a compendium of genes related to tumor invasiveness, we systematically searched published studies and adopted a 24-gene expression signature derived from a comprehensive pan-cancer analysis using the “extreme value association” algorithm, which identifies sets of genes whose coordinated overexpression indicates the presence of a advanced-stage phenotype [18] (Additional file 7: Data S1). The signature includes COL11A1, POSTN, EPYC, ASPN, COL10A1, THBS2, FAP, LOX, SFRP4, INHBA, MFAP5, GREM1, COMP, VCAN, COL5A2, COL5A1, TIMP3, GAS1, TNFAIP6, ADAM12, FBN1, SULF1, COL1A1, and DCN. The invasiveness scores were computed from RNA sequencing of each bulk sample using the gene set variation analysis (GSVA) algorithm in the *GSVA* package [19], an unsupervised gene set enrichment method that computes an enrichment score by integrating the collective expression of a given gene set relative to the other genes in the sample. It has been reported that GSVA outperforms single cell gene set enrichment analysis (ssGSEA) when measuring the signal-to-noise ratio in differential gene expression and differential pathway activity identification analyses because GSVA includes normalization of gene expression aimed at reducing the noise of the data [20]. The distribution patterns of the invasiveness score in different patients were plotted using the *pheatmap* package. Afterwards, we classified patients into high- and low-invasiveness groups based on an optimal cutoff value of the invasiveness score determined by log-rank test of recurrence-free survival (RFS) or overall survival (OS). The cutoff value was obtained with the assistance of *survminer* package, which finds the optimal cut point of one or multiple continuous variables at once that correspond to the most significant relation with survival outcome (RFS or OS here), using the maximally selected rank statistics. Considering the natural discrepancy in prognosis of different cancer types, we determined the cutoff value of the invasiveness score in each cancer separately. Results are presented with the *forestplot* package.

### Differential expression and functional enrichment analysis

Differentially expressed genes (DEGs), miRNAs, proteins, were identified between high- and low-invasiveness score groups across cancer types using the package *limma*. All the p-values generated from multiple tests for high throughput data, including differentially expressed gene, miRNA, protein, methylation, somatic mutation, and copy number variation analyses, as well as functional enrichment analyses, were adjusted as false discovery rate (FDR). |Log (fold change) (log FC)| > 0.5 and FDR < 0.05 were considered cutoff criteria to screen for differential expression. Functional enrichment analyses of the detected DEGs were performed with the *clusterProfiler* package [21]. Gene Ontology (GO) and Kyoto Encyclopedia of Genes and Genomes (KEGG) terms were identified with a strict cutoff of FDR < 0.05. We also obtained gene sets that represented different biological pathways from several publications, including tumor-infiltrating lymphocytes (TILs) [22], immune cytolytic activity (CYT), interferon (IFN) response [23], hypoxia score [3], and the immune score based on the ESTIMATE, which calculates immune and stromal scores to predict the infiltration of non-tumor cells by analyzing specific gene expression signatures of immune and stromal cells [24] (Additional file 7: Data S1). ssGSEA was used to quantify the enrichment levels of these signatures in each tumor sample [25].

### Comparison of somatic mutations and CNV

Comparison of the somatic mutations and gistic-identified CNVs between high- and low-invasiveness score groups across cancer types was tested using the Kruskal–Wallis test, where p-values < 0.01 after adjustment for mutational frequency were considered statistically significant. Results were generated with the oncoplot function in the *maftools* package.

### Preprocessing and analysis of DNA methylation data

We employed the TCGA DNA methylation data obtained by the Illumina Human Methylation450 BeadChip array, which contains 485,577 probes (396,066 after filtering invalid probes) covering 99% of RefSeq genes. The methylation levels of each probe were quantified as β-values, which are the ratios of the intensities of methylated and unmethylated alleles. 5′-C-phosphate-G-3′ (CpG) methylation data between high- and low-invasiveness score groups were normalized and compared with the CHAMP pipeline [26]. The algorithm used for differentially methylated CpG sites is similar to that used in the DEG analysis, and the threshold was set as FDR < 0.05 and an absolute ∆β-value > 0.2. A gene was considered to be differentially methylated if there was at least one differentially methylated CpG in its promoter region.

### Statistical analysis

All statistical analyses were conducted using R software (Version 3.5.3; R Foundation for Statistical Computing, Vienna, Austria). We performed the Pearson correlation test and calculated the coefficient (r) with corresponding *p* value when necessary, considering |r| > 0.3 and p < 0.05 as strong correlation. We employed several R packages for data visualization, including *circlize*, *ggpubr*, *igraph*, and *networkD3*. Student’s *t*-test and the Wilcoxon test were used to compare continuous variables between the high- and low-invasiveness score groups, while Chi-square and Fisher’s exact tests were used for categorical variables when appropriate. Log-rank tests and Kaplan–Meier survival curves visualized by the *ggplot2* package were used to compare survival between different populations. A univariable Cox proportional risk analysis was performed to test the prognostic value of the invasiveness score. The p-values were all two-sided. In the Chi-square test, Fisher’s exact test, log-rank test, Cox analysis, and correlation test, p-values < 0.05 were considered statistically significant.

## Result

### Classification and validation of the invasiveness score groups based on a 24-gene signature

**Fig. 1:**
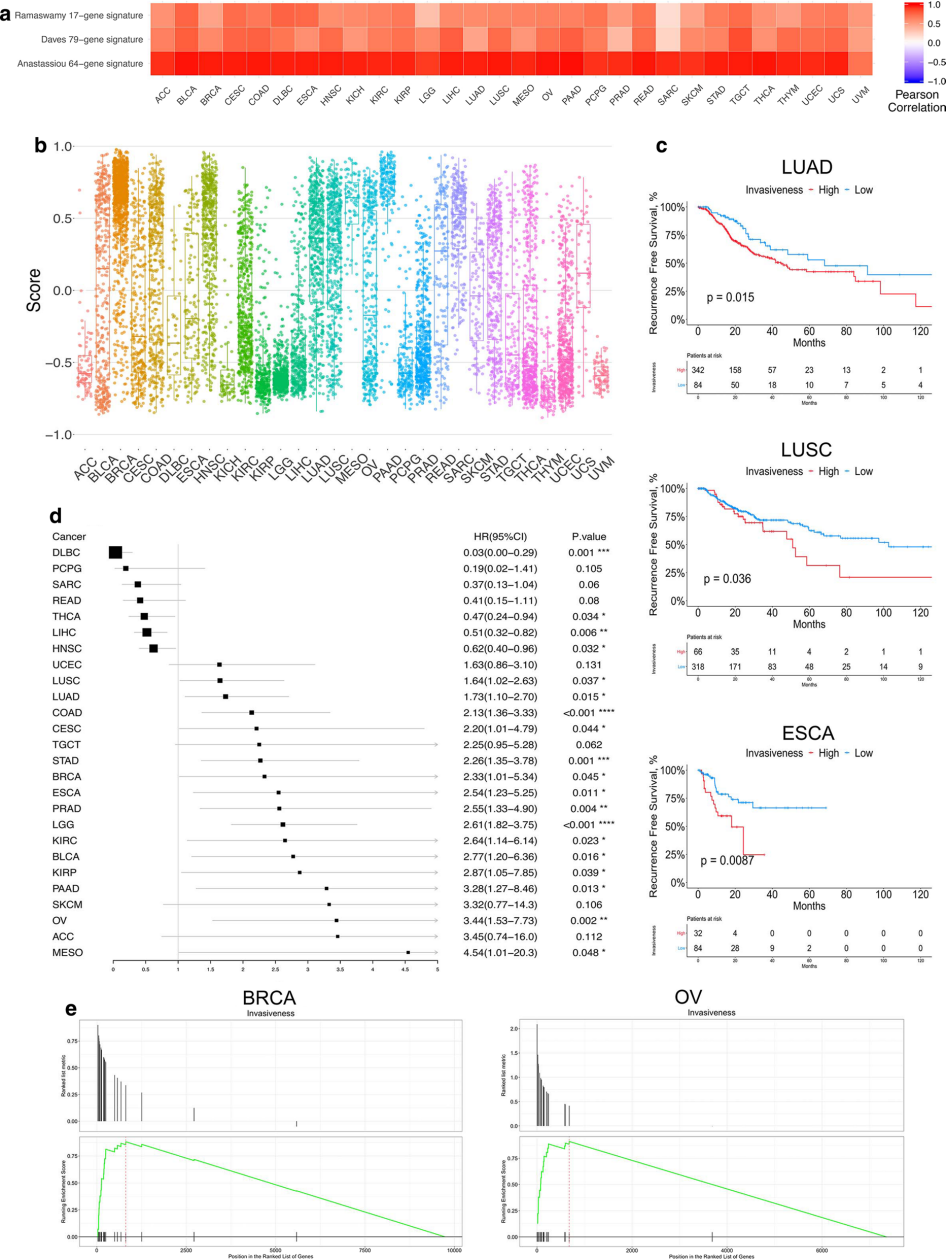
**a** Spearman’s correlation of the invasiveness score between this 24-gene signature and the other two gene signatures across 30 cancer types. **b** The distribution of the invasiveness score across 30 cancer types. Within each group, the scattered dots represent the invasiveness score of each patient. The lines in the boxes represents the median value. The bottom and top of the boxes are the 25th and 75th percentiles (interquartile range). The whiskers encompass 1.5 times the interquartile range. **c** Kaplan–Meier curves shows that high-invasiveness score group is associated with worse RFS in LUAD, LUSC, and ESCA cohort. **d** The prognostic value of invasiveness score in each cancer type, shown in the forest plot with corresponding hazard ratio (HR) and 95% confident interval (95% CI). **e** Gene set enrichment analysis (GSEA) based on mass proteomic spectrum data indicated that the proteins in the invasiveness-signature were significantly enriched in the high-invasiveness score group in BRCA and OV

The study design is shown in Additional file 2: Figure S1. To investigate the invasiveness-related genes across human tumors, we focused on a 24-gene expression signature [18] (Additional file 7: Data S1) and calculated the invasiveness score by GSVA based on this signature for each cancer patient enrolled in this study (Additional file 7: Data S2). Another three invasiveness gene signatures, including 64, 17, and 79 genes (Additional file 7: Data S1) [27–29], were also screened for initial analysis; however, by investigating the prognostic value of the 4 candidate gene signatures in LUAD patients, our 24-gene signature exhibited the best prognostic value as an indicator of tumor invasiveness (Fig. 1c, Additional file 7: Figure S2A). The robustness of this invasiveness-associated signature has also been verified in several previous independent studies [30–32]. The GSVA score based on this signature was highly correlated with the invasiveness score for the other three signatures in the 30 cancer types included in TCGA (Fig. 1a), further suggesting the reliability and practicality of this 24-gene signature to define the tumor invasiveness level in multiple cancer types.

The invasiveness score exhibited distinct distribution patterns in different cancer types (Fig. 1b). For example, the median (interquartile range) was 0.752 (0.651–0.843) for pancreatic cancer (PAAD); for thymoma (THYM), the value was much lower: − 0.678 (− 0.757 to − 0.560). These results suggest that invasiveness-related genes are significantly upregulated in malignancies such as PAAD and BRCA, thus leading to the increased score. Considering the relatively poor prognosis of patients with PAAD compared with other tumor types such as THYM [1] (5 year survival rate in TCGA: 0.225 for PAAD and 0.926 for THYM) it could be inferred that the 24-gene signature and corresponding score precisely reflect the degree of malignancy of the cancer, which is consistent with our speculation above.

In each cancer type, we classified patients into high- and low-invasiveness groups based on the invasiveness score and RFS. Although overall survival (OS) is universally recognized as the gold standard when assessing prognostic information or measuring treatment effects in clinical research, the complexity of cancer death, including invasion, recurrence, and metastasis, still limits the practicality and reliability of OS in the estimation of cancer progress and prognosis. Therefore, considering the superiority of RFS compared with OS in such circumstances [33, 34], we selected RFS in this study to investigate the association between invasiveness and cancer survival. Acute myeloid leukemia (LAML), bile duct cancer (CHOL), and glioblastoma (GBM) were excluded because of the inaccessibility of their RFS data and the remaining 30 cancer types, which included a total of 9356 patients, were finally enrolled for subsequent analysis. The correlation between the AJCC TNM staging system and our invasiveness group classification is shown in Additional file 7: Figure S3A. Patients in the high- and low-invasiveness groups showed distinct enrichment of the 24 genes in our signature (Additional file 7: Figure S2B), and high-invasiveness scores were consistently associated with worse prognosis in the majority of the 30 cancer types, including LUAD (hazard ratio [HR]: 1.73 (1.10–2.70), p = 0.015), LUSC (HR: 1.64 (1.02–2.63), p = 0.037), and esophageal carcinoma (ESCA; HR: 2.54 (1.23–5.25), p = 0.011; (Fig. 1c, d and Additional file 3: Figure S4), indicating the robust prognostic value of our score-originated invasiveness group classification in both RNA sequencing and gene-chip data. However, interestingly, high-invasiveness scores were found to be significantly associated with better prognosis in only four cancer types: diffuse large B cell lymphoma (DLBC), thyroid carcinoma (THCA), liver hepatocellular carcinoma (LIHC), and head/neck squamous cell carcinoma (HNSC) (Additional file 3: Figure S4).

We externally validated the prognostic value of our invasiveness score group classification in non-small-cell lung carcinoma patients from our institution, as well as three independent GEO datasets of LUAD, LUSC, and ESCA (Additional file 4: Figure S5). Besides, similar results were also obtained from 10 independent datasets from PRECOG database, including LUAD, COAD, STAD, and so on. Unfortunately, RFS data was not available in PRECOG, therefore we had to perform our analyses based on OS here (Additional file 4: Figure S5). Moreover, to further explore whether we could reach similar results at a post-transcriptional level, we performed gene set enrichment analysis (GSEA) in 102 BRCA patients and ten ovarian cancer (OV) patients whose mass proteomic spectrum data were available in the CTPAC [9, 10]. The expression data of 9733 and 7625 proteins was available for BRCA and OV patients, respectively. Based on the invasiveness group determined by RNA-sequencing data, the 24-gene signature, which corresponds to 24 proteins, was also enriched in the high-invasiveness score group for both cancer types (FDR = 0.00261 for BRCA and 0.0021 for OV; Fig. 1e). This phenomenon could be explained by the high correlations between mRNA and protein expression levels in a patient. Taken together, our analyses integrating RNA-sequencing, RNA-chip, proteomic, and survival data support the validation of our invasiveness score group classification across different cancer types.

### Multidimensional characterization of invasiveness-related genomic features across multiple cancer types

**Fig. 2:**
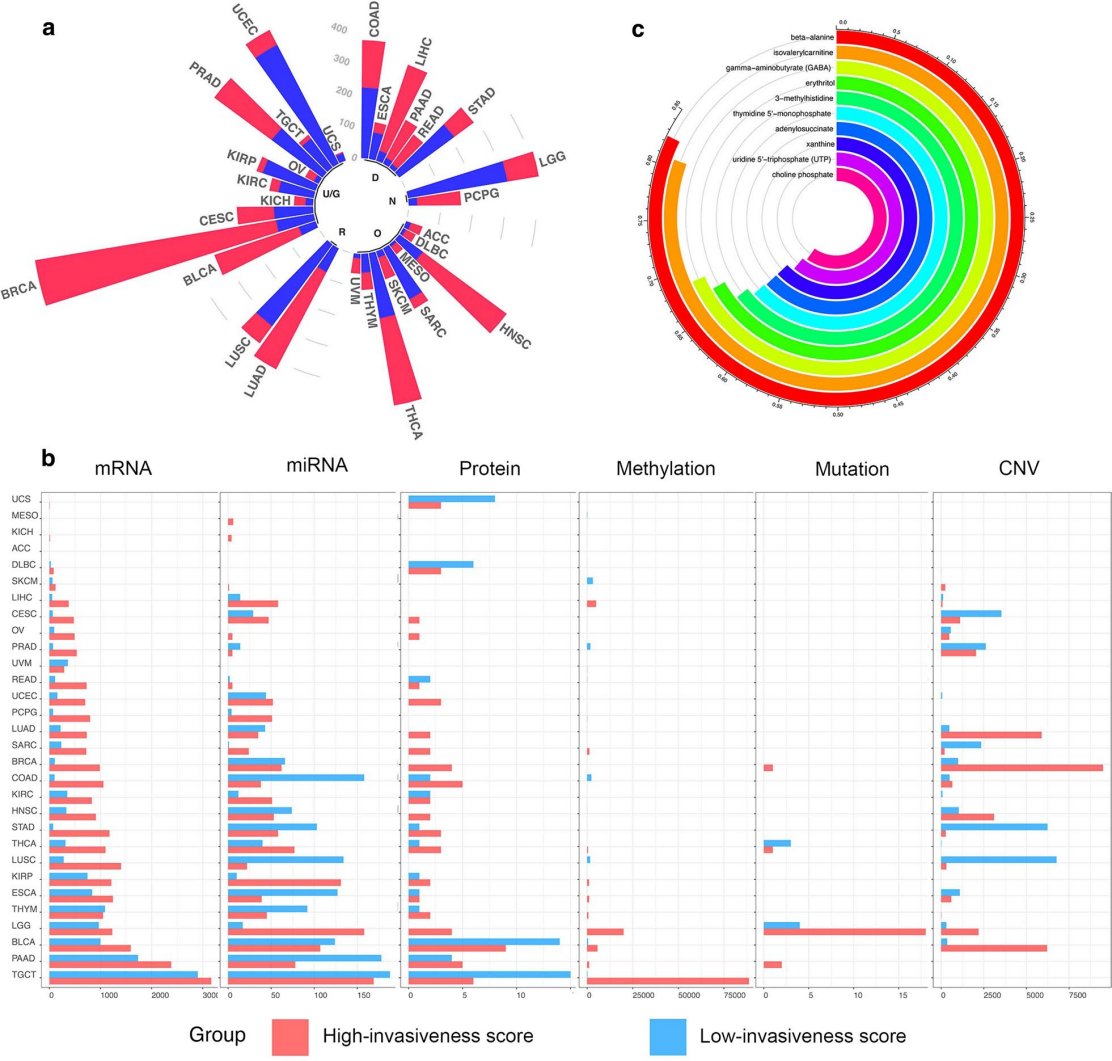
**a** The circular bar plot displays the percentages and numbers of samples in high- and low-invasiveness score group across multiple cancer types. Red bar represents high-invasiveness group while blue represents low. The cancers are also annotated with the physiology systems they belong to: ‘R’, Respiratory system; ‘U/G’, Urinary system and Genital system; ‘D’, Digestive system; ‘N’, Nervous system; ‘O’, Other systems. **b** Relative abundance and numbers of multidimensional significant discrepant invasiveness-associated molecular features. **c** The circos plot exhibits invasiveness-related metabolites in BRCA patients. The length of each arc indicates the Spearman correlation between the invasiveness score and a series of metabolites. Top ten negatively-correlated metabolites with the highest |r| were displayed in the plot

The proportions of high- and low-invasiveness score groups varied greatly among different cancer types (Fig. 2a). For example, the high-invasiveness score group included 86.36% (133 of 154) of PAAD and 86.44% of BRCA patients. By contrast, only 20.57% (29 of 141) and 47.79% (54 of 113) of KIRC and THYM patients, respectively, were classified into the high-invasiveness score group. These results demonstrate the distinct overall invasiveness of different cancer types, consistent with the results shown in Fig. 1b.

To systematically outline the genomic characteristics of the invasiveness of patient cancers, we performed a multidimensional comparison of six types of molecular features between the high- and low-invasiveness groups in 30 cancer types: mRNA-sequencing (58,387 genes), mature miRNA (approximately 2000 miRNAs), protein expression (223 proteins), DNA methylation (396,066 probes covering > 20,000 genes), somatic mutation, and CNV data. Significantly different features were identified (Fig. 2b).

Differential genomic features between the high- and low-invasiveness groups exhibited varied distribution across the 30 cancer types, which could be explained by the different tumor characteristics and varied sample sizes. For instance, only six DEGs were detected in uterine carcinosarcoma (UCS), while 6069 were detected in testicular cancer (TGCT). We failed to identify differentially expressed miRNAs in adrenocortical cancer (ACC), large B-cell lymphoma (DLBC), rectal cancer (READ), melanoma (SKCM), and UCS but identified 180 in bladder cancer (BLCA). Alterations in DNA methylation probes ranged from one in kidney chromophobe (KICH) and colon cancer (COAD) to 88,772 in TGCT. No significantly differentially expressed proteins were identified after FDR adjustment, thus we just displayed the proteins with an unadjusted P-value < 0.05 in Fig. 2b. In terms of differentially mutated genes, only four sites in BRCA, 22 in lower grade glioma (LGG), two in PAAD, and four in THCA were found. However, a large number of CNV alterations were identified; the largest number was 10,484 in BRCA (Fig. 2b, Additional file 7: Data S3).

Remodeling of cellular metabolism appears to be a consequence and possibly a cause of oncogenic transformation in human cancers. A study by Tang et al. analyzed 25 breast cancer patients (23 fully analyzed by TCGA) by chromatography/mass spectrometry and quantitated 399 identifiable metabolites. We explored the correlation between these metabolites and our invasiveness score in BRCA cancer patients and identified 40 metabolites, such as β-alanine (r = − 0.825) and isovalerylcarnitine (r = − 0.808) [11], that were significantly negatively correlated with the score (Fig. 2c). However, only two metabolites, glutamine (r = 0.457) and myristate (r = − 447), were significantly positively correlated with the score (Additional file 7: Figure S3B).

### Integrated correlation analysis of drug response and DEGs across tumor types

**Fig. 3:**
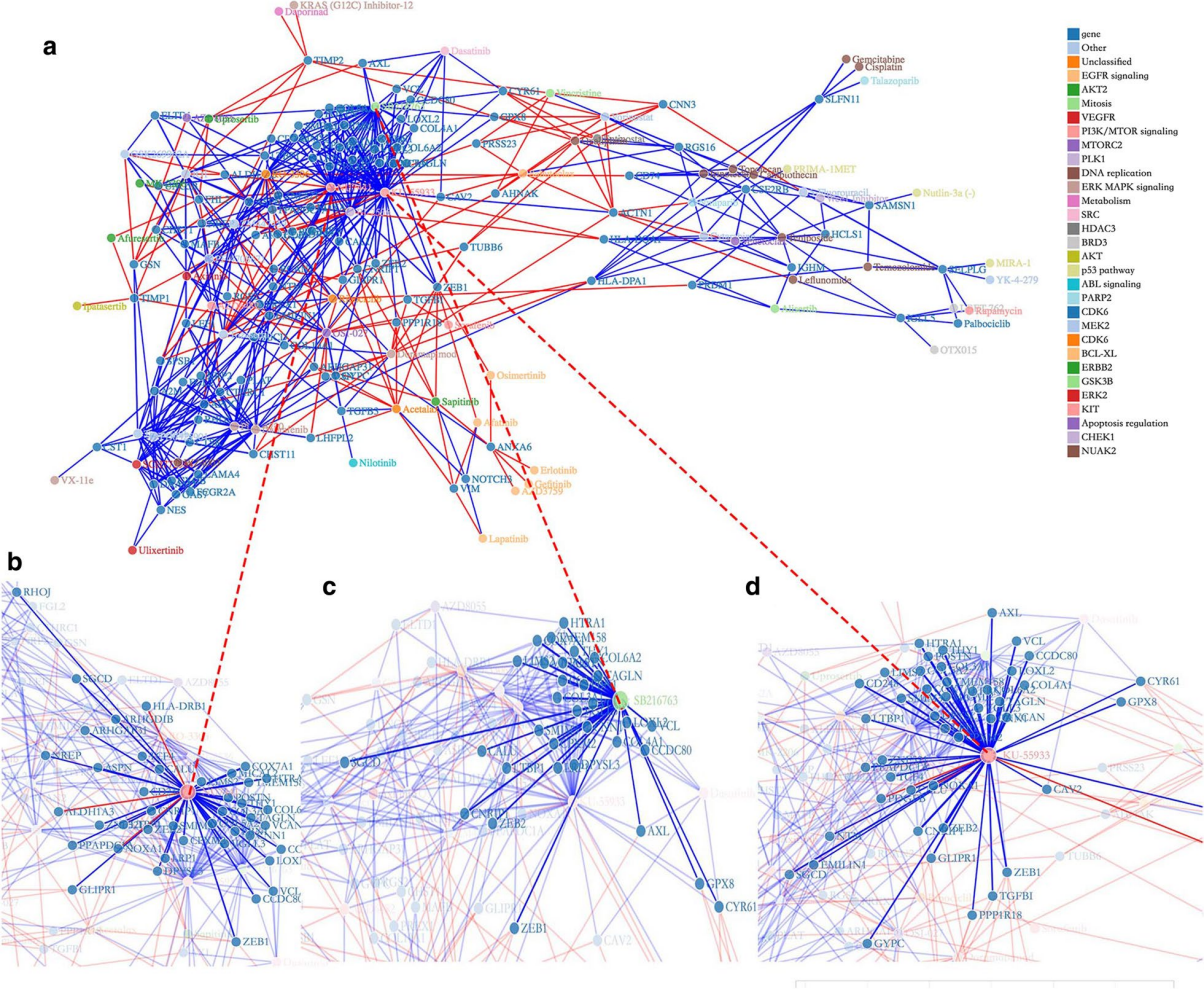
**a** The network exhibits the correlation between the drug sensitivity in cancer cell lines from GDSC and the expression level of 685 invasiveness-associated DEGs across cancer types. Dark blue dots represent genes, while dots with other colors represent the anti-cancer drugs targeting different biological pathways. Red lines indicate positive correlation and blue lines indicate negative. **b**–**d** Genes significantly correlated with three important anti-cancer drugs—NU7441 (**b**), SB216763 (**c**), and KU-55933 (**d**)—were highlighted

Given the previously reported association of drug resistance with invasiveness and the epithelial–mesenchymal transition (EMT) [35–37], we speculated that the DEGs between the high- and low-invasiveness score groups might serve as therapeutic targets. Therefore, we comprehensively analyzed correlations between the drug sensitivity data from GDSC and the expression level of 685 genes, which were significantly differentially expressed in at least ten cancer types, in cancer cell lines. Cell lines not derived from the 30 cancer types in TCGA were excluded. Finally, the RNA sequencing data of 435 cancer cell lines and their sensitivity to 169 anti-cancer drugs, presented as AUC (see “Methods and materials”), were used for subsequent analyses (Additional file 7: Data S4). These drugs target several important biological processes, including metabolism, apoptosis, chromatin histone methylation, DNA replication, EGFR signaling, and the p53 pathway. As shown in Fig. 3a, the network depicts the complicated correlations among the anti-cancer drugs and 104 of 685 invasiveness-related DEGs that were strongly correlated (|r| > 0.3) with cell line sensitivity (AUC) to at least four drugs [an interactive version of Fig. 3a is also provided, which is available at http://www.datapredictionzc.com/invasiveness_drugresponse, enabling further exploration of this complex network]. For example, COL3A1, a key gene in the constitution of the extracellular matrix (ECM), is significantly upregulated in the high-invasiveness score group in 26 cancer types. Overexpression of COL3A1 mRNA in 435 cell lines was linked to higher sensitivity to four anti-cancer drugs, including the ATM kinase inhibitor KU55933 (r = − 0.599). It has been reported that overexpression of COL3A1 is correlated with worse prognosis in many cancers [38, 39]. A2M, which is involved in the Fas apoptotic signaling pathway, was upregulated in 18 cancers in the high-invasiveness score group and negatively correlated with sensitivity to eight drugs, such as Axitinib (r = − 0.503) and Dabrafenib (r = − 0.494). Overexpression of ACTN1 in the high-invasiveness score group was detected in 15 cancer types and suggested potential resistance to eight drugs, including Oxaliplatin and Leflunomide, both of the which target DNA replication (r = 0.401 and r = 0.349, respectively).

In terms of drugs, we identified the DNA-PK inhibitor NU7441, the GSK3 inhibitor SB216763, and KU55933 as three “core nodes” in the network since they were significantly correlated with the expression of 77, 91, and 110 invasiveness-related DEGs in our analysis (Fig. 3b–d). Taken together, our results demonstrate the extensive interactions between drug responses and invasiveness-related genomic features, highlighting the potential of our invasiveness score as a predictor of anti-cancer therapeutic effects.

### Application of the invasiveness score in assessment of other biological processes

**Fig. 4:**
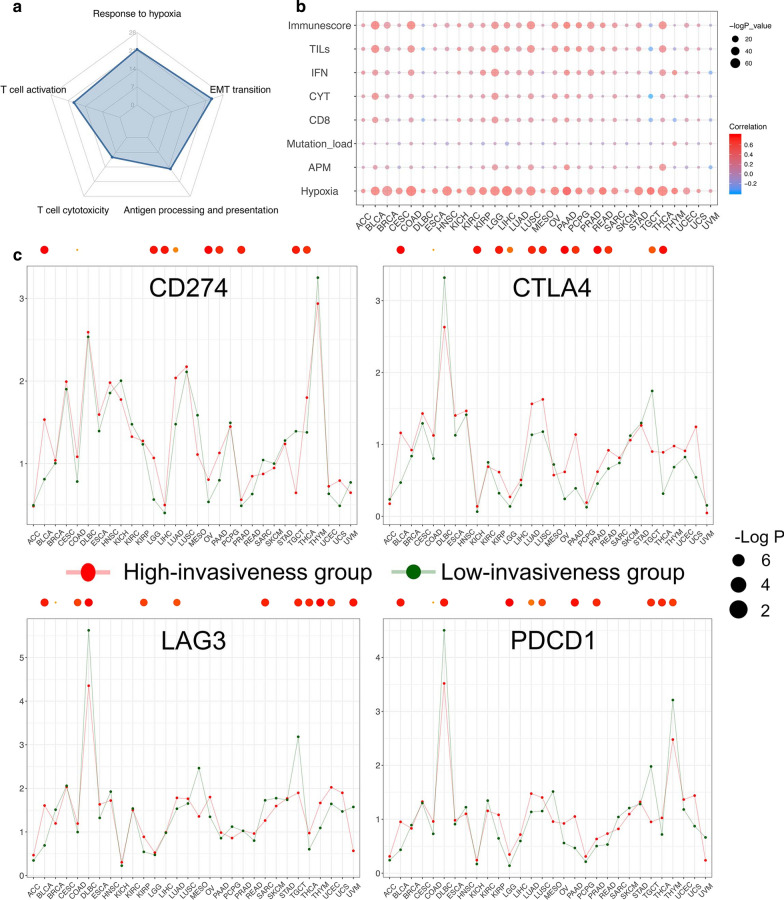
**a** The radar plot exhibits several GO biological pathways that were significantly enriched in multiple cancer types based on the invasiveness-related DEGs. The number of cancers of each biological pathway is shown as the position of the corresponding dot in each spoke. **b** Correlation between the invasiveness score and the ssGSEA score of several pathways, as well as the mutation load, across 30 cancer types. Dot size is proportional to the −log P-value of correlation; color indicates magnitude of correlation (r value). **c** Comparison of the 4 immune-checkpoint molecules’ expression level in high- and low-invasiveness group across multiple cancer types. The points connected by lines represent the mean value of the expression level in each group across different cancer types, whereas the significantly differential expression is annotated with a red dot on the top of plot. Dot size are proportional to the −log P-value of t-test

To better understand the biological processes globally dysregulated with different invasiveness statuses, we conducted GO enrichment analyses of the DEGs in each cancer type and screened the pathways that were significantly enriched in at least ten cancer types (Additional file 7: Data S5). Studies have shown that the EMT plays an important role in cancer invasiveness, metastasis, and drug resistance [36, 40, 41], which is consistent with enrichment of “Epithelial-to-mesenchymal transition” [GO: 0001837] in 25 cancer types (Fig. 4a). Interestingly, we noticed that “response to hypoxia” [GO: 0001666] and a series of immune-related pathways like T cell activation (GO: 0050863) were also generally enriched across multiple tumor types (Fig. 4a). Therefore, we performed a more focused analysis to investigate the association between our invasiveness score and cancer patient immunity or hypoxia status.

The “immune score” and “hypoxia score” were calculated by ssGSEA based on the signatures proposed by Yoshihara et al. [24] (Additional file 7: Data S1). As depicted in Fig. 4b, we observed a consistent and strong positive correlation between the invasiveness score and the hypoxia score across all cancer types, indicating the potential of combining anti-hypoxia drugs with conventional anti-cancer therapies in patients with higher invasiveness scores. In terms of immunity, invasiveness was positively correlated with the immune score and diverse immune signatures across several cancer types, including the expression of CD8A, TIL infiltration, CYT, the IFN response [42], and an expression signature of antigen processing and presenting machinery (Fig. 4b). However, the correlation between the invasiveness score and the mutation load, a well-recognized biomarker for immunotherapy, was weak. In addition to the immune-related pathway (gene set), we found significant overexpression of four immune checkpoint genes in the high-invasiveness group in various cancers, including BLCA and LUAD (Fig. 4c). These results suggest that highly invasive tumors tend to exhibit an immune-stimulatory tumor microenvironment, and the invasiveness score might serve as a new predictive biomarker for the response of cancer patients to immune checkpoint inhibitor treatment.

### Invasiveness-associated differential DNA methylation, miRNA expression, and CNV

Gene expression is regulated by various factors, such as DNA methylation, miRNAs, somatic mutations, and CNVs. Thus, we performed an integrative assessment of the associations between invasiveness-associated DEGs and multidimensional molecular alterations to determine the drivers of genomic dysregulation.

**Fig. 5:**
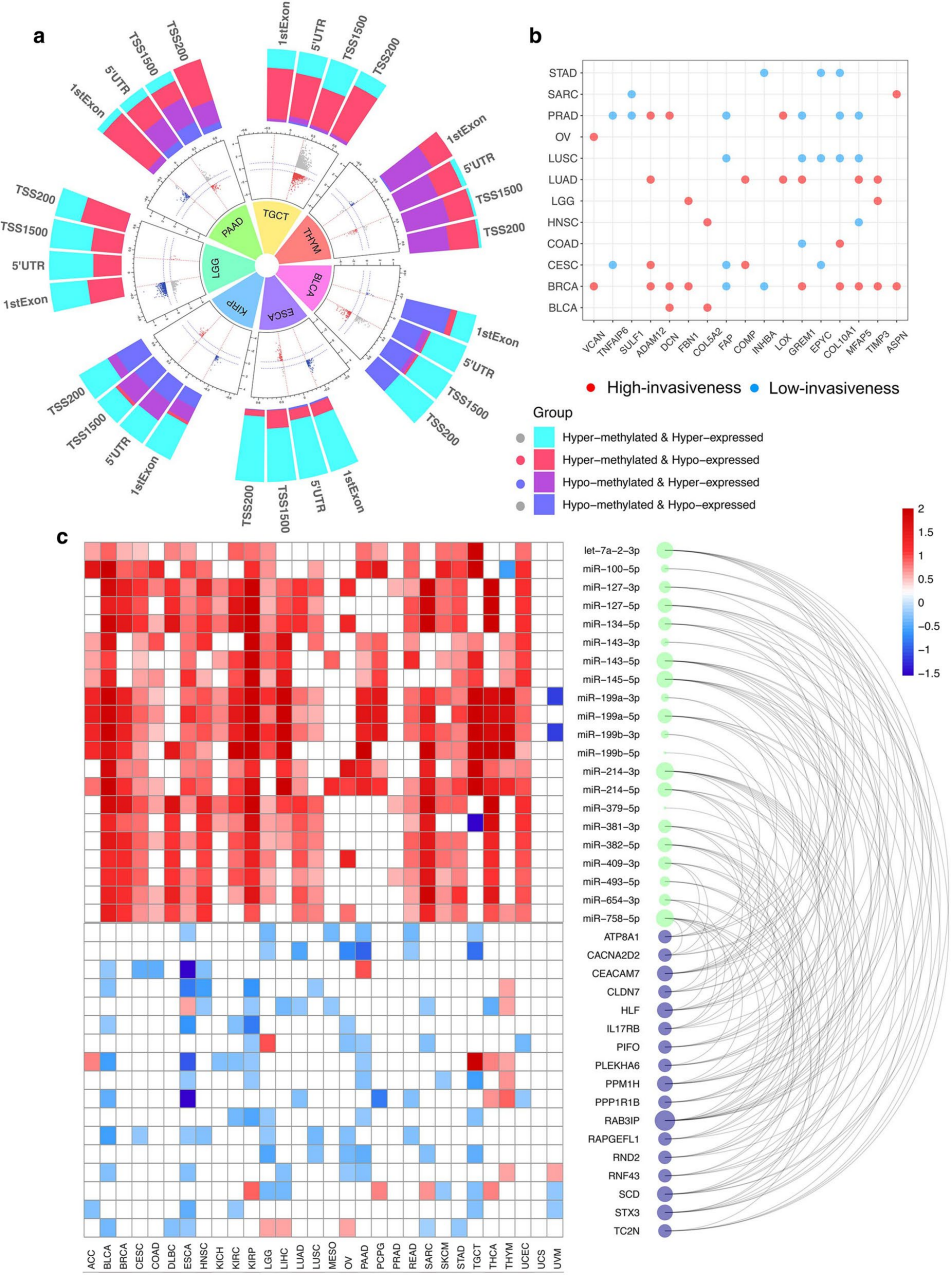
**a** Inner ring: scatter plot of mean methylation difference (shown as delta beta value) versus expression difference (shown as log Fold change) in 7 cancer types. Each point represents a CpG-gene pair. Outer ring: Bar plot exhibited the distribution of differentially methylated and expressed genes across gene regions (promoter only: TSS1500, TSS200, 5′-UTR, 1st exons) in different cancer types. **b** Invasiveness-associated copy number variations (CNVs) across twelve cancer types. The results show significantly higher CNV frequency in high-invasiveness score group (red), and lower frequency in low-invasiveness score group (blue). **c** Common upregulated miRNAs and downregulated genes in high-invasiveness score group across multiple cancer types. The arc plot (right) shows the targeting relationship (thin lines) between miRNAs (green dots) and genes (purple dots). The heatmaps (left) exhibits the log Fold change (high-invasiveness vs. low-invasiveness) of each miRNA and gene in different cancer types, where red cell represents significantly upregulated while blue represents significantly downregulated and white represents no-significance

DNA methylation was the first epigenetic abnormality recognized in human cancer and is a ubiquitous feature of carcinogenesis [43], where hypermethylation generally leads to gene silencing and hypomethylation results in overexpression. The distribution of differentially methylated CpGs between the high- and low-invasiveness score groups in the different cancer types is presented in Additional file 5: Figure S6. In this study, we focused on methylation of the gene promoter region including TSS200, TSS1500, 3′-UTR, and 1st-Exon (Fig. 5a), since differentially methylated genes (DMGs) are commonly defined according to their promoter methylation status [44, 45]. The genes were classified into four groups based on the intersection between invasiveness-associated DMGs and DEGs: hypermethylated and upregulated (hyper-up), hypermethylated and downregulated (hyper-down), hypomethylated and upregulated (hypo-up), and hypomethylated and downregulated (hypo-down) (Fig. 5a, Additional file 7: Data S6). Considering the nature of DMGs and DEGs, we focused on genes in the hyper-down and hypo-up groups in downstream analyses. For example, in ESCA, 83 genes were hypermethylated and downregulated, while 140 were hypomethylated and upregulated. However, these differentially methylated and expressed genes (DMEGs) exhibited few generalities across tumor types as no identified DMEG existed in even at least three cancers. We also investigated whether somatic mutations and CNVs were associated with the invasiveness group independent of tumor origin. Only a few differentially mutated genes were detected between the high- and low-invasiveness groups (Fig. 2b). Despite a large number of significant invasiveness-related CNVs, the results still lacked generality and could be primarily attributed to CNVs specific to certain tumor types (Fig. 5b). These results suggest that abnormal methylation, mutation, and CNV of a specific gene might not be a major driver of the dysregulation of invasiveness-related genes in human cancer.

We also investigated the intersection between invasiveness-related DEGs and differentially expressed miRNAs across multiple cancer types based on the miRNA-gene targeting relationship predicted by miRWalk. As shown in Fig. 5c, 21 miRNAs were significantly overexpressed in the high-invasiveness score group in at least 16 cancer types, while 17 genes, which were potentially regulated by these miRNAs, were downregulated in at least five tumors (Additional file 7: Data S7). For instance, members of the miR-199 and miR-214 families, which have been reported to cooperatively regulate the EMT in triple negative breast cancer [46] and promote invasion and metastasis of melanoma [47], were upregulated in 15 cancers, including BLCA, BRCA, and ESCA, while their target genes, such as CEACAM, were downregulated. Downregulation of CEACAM is considered a predictive marker of rectal cancer recurrence [48], and HLF serves as a driver of hepatocellular carcinoma [49]. The heatmaps in Fig. 5c demonstrate that the logFC between the high- and low-invasiveness score groups of these target genes tended to be the opposite of miRNAs, indicating the important role miRNAs play in the regulation of invasiveness.

### Investigation of invasiveness-associated genomic alterations and drug sensitivity in ESCA

**Fig. 6:**
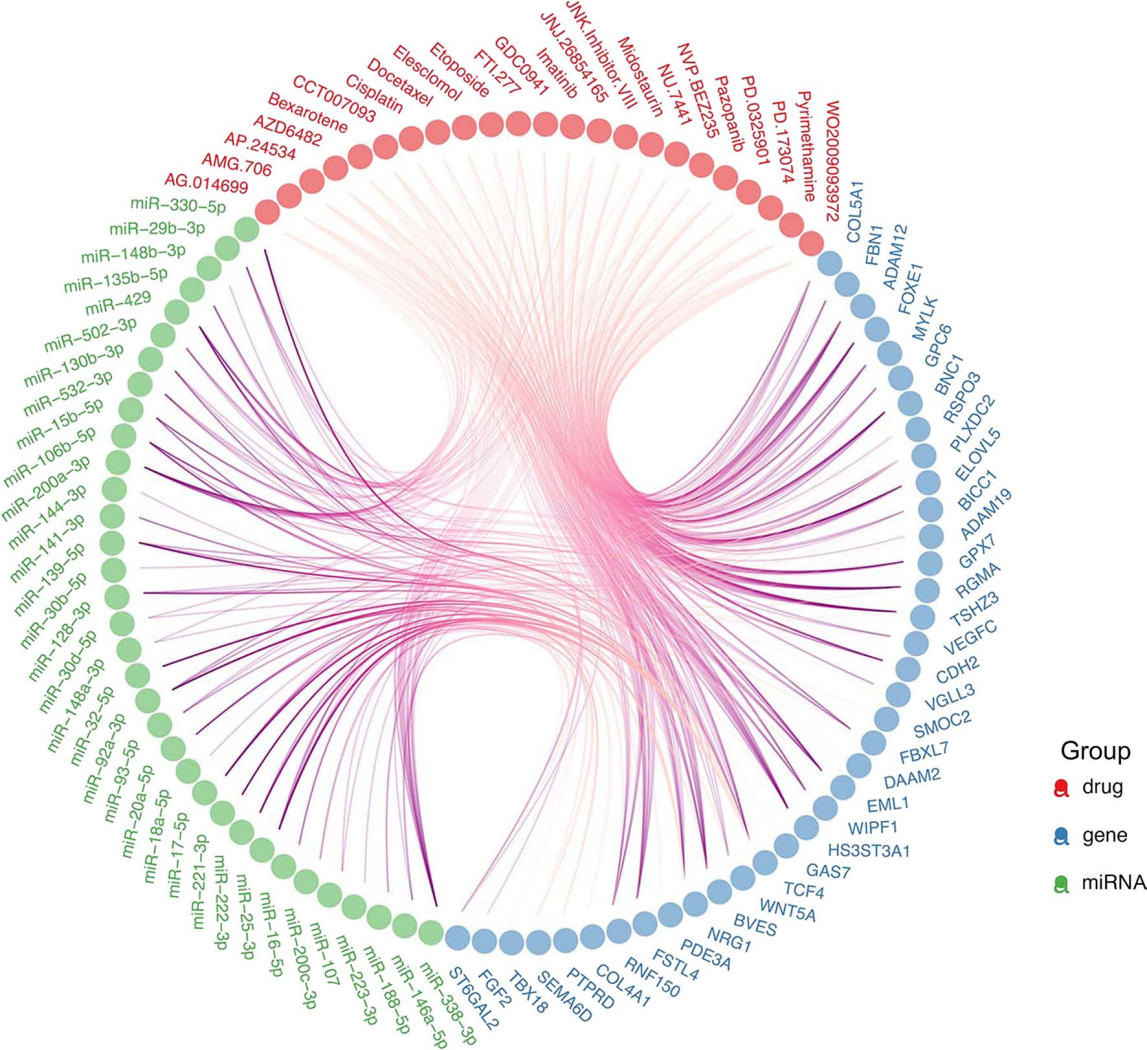
Intersection of invasiveness-related mRNAs, DNA methylation, miRNAs and response in ESCA. Upregulated genes (blue dots) in high-invasiveness group were all hypo-methylated and regulated by multiple downregulated miRNAs (green dots). The expression levels of these genes significantly correlated with the IC50 to various kinds of anti-cancer drug (red dots, |r| > 0.3, p < 0.05), while that of miRNAs displayed opposite correlation with the same drug. The pink lines represent the targeting relationship between miRNA and genes, or the correlation between miRNA/gene and drug response

To further explore the association between the invasiveness status of a tumor and sensitivity to anti-cancer treatment, we performed a thorough and integrated analysis to assess correlations among multidimensional invasiveness-related molecular features and drug response data predicted by Geeleher et al. based on a novel algorithm [15]. We focused on ESCA as an example since ESCA is one of the most focused cancer types in our department of thoracic surgery, and exhibits the most typical mutual regulation of RNAs, miRNAs, and DNA methylation. A total of 38 genes were significantly upregulated in the high-invasiveness score group, and all of which were hypomethylated and regulated by several downregulated miRNAs (Fig. 6). The expression levels of these genes were significantly correlated with the IC_50_ values of various anti-cancer drugs (|r| > 0.3, p < 0.05), while miRNAs displayed opposite correlations with the same drug (Additional file 7: Data S8). For example, ADAM12, a biomarker of cancer stem cell phenotype which has been reported to promote esophageal squamous cell carcinomas invasion [50, 51], was upregulated in high-invasiveness ESCA patients (logFC = 2.01, FDR = 2.92 × 10^−22^), and showed hypomethylation in the promoter region (logFC, or delta-β = − 0.25, FDR = 0.0005). The expression level of ADAM12 was negatively correlated with the response to 20 anti-cancer drugs, inclu-ding the tyrosine kinase inhibitors Imatinib and Pazopanib (r = − 0.701 and − 0.598, respectively), the angiokinase inhibitor AMG.706 (r = − 0.626), the mitosis inhibitor Docetaxel (r = − 0.553), and Cisplatin, which interferes with DNA replication (r = − 0.531). Three miRNAs that target the 3′-UTR regions of ADAM12 exhibited significant downregulation in the high-invasiveness score group (miR-130b-3p: logFC = − 0.535, FDR = 0.041; miR-130b-3p: logFC = − 0.509, FDR = 0.005; miR-502-3p: logFC = − 0.713, FDR = 1.554 × 10^−04^), and the miRNAs positively correlated with drug responses (resistance) which have negative correlations with ADAM12, such as miR-30b-5p with Imatinib and Docetaxel (r = 0.418 and r = 0.404, respectively) and miR-502-3p with Imatinib and Cisplatin (r = 0.484 and r = 0.470, respectively). Moreover, a similar gene-methylation-miRNA-drug regulatory network was observed in genes upregulated in low-invasiveness score ESCA patients (Additional file 6: Figure S7, Additional file 7: Data S9). Taken together, our findings demonstrate the complicated regulatory mechanism of tumor invasiveness in multiple dimensions, and present several potential biomarkers and therapeutic targets for future research.

## Discussion

Tumor invasiveness reflects a series of biological changes that contribute to tumorigenesis, progression, metastasis, and response to anti-cancer therapy. Therefore, a comprehensive genomic analysis focusing on the comparison of cancer patients characterized by different invasiveness statuses will be important to guide more precise and personalized anti-cancer therapeutic strategies [18, 52]. In this study, we first demonstrated the robustness of a 24-gene signature that defines malignancy and tumor invasiveness across 30 cancer types in TCGA, and then classified tumor patients into high- and low-invasiveness score groups based on the invasiveness score in each cancer. The prognostic value of the invasiveness score was determined and externally validated in several independent cohorts. Moreover, by integrating multi-omics data, we provided an integrative view of invasiveness-associated dysregulated molecular features and investigated their mutual interfering relationships and correlations with drug responses, thus depicting the complex regulatory network of tumor invasiveness in multiple dimensions. The major results for each result subsection and some heuristic choices used for the criteria for common molecular alteration in different datatype in this study were summarized in Additional file 1: Tables S1 and S2.

It has been hypothesized that as carcinomas arising from epithelial tissues progress to higher pathological grades of malignancy, reflected in local invasion and distant metastasis, the associated cancer cells typically develop alterations in their shape as well as in their attachment to other cells and to the ECM [2]. This complex process can be considered a consequence of the combined effect of the EMT [53, 54], the presence of activated fibroblasts in the reactive desmoplastic stroma, and the tumor microenvironment, which are regulated by accumulated genetic and epigenetic alterations. The interruption of any one or more of these steps could potentially inhibit the development of tumor invasiveness [55].

The 24-gene signature proposed by Kim et al. used in this study integrates critical regulator genes in this cascade, including THBS2 and INHBA, which are involved in TGF-β signaling and facilitate fibroblast-mediated collagen gel contraction, resulting in ECM remodeling and tumor invasiveness [56, 57]. Hence, it is consistent with previous studies that in most cancer types (23 of 30), a higher invasiveness score was associated with worse RFS, and its prognostic value was further validated in several external cohorts from GEO, PRECOG, and our institution. However, in a few cancers, such as DLBC, THCA, LIHC, and HNSC, we observed opposite results, which may be a result of the different origin, biological characteristic, and microenvironment of specific cancer types. It is widely accepted that one gene might play diverse roles in different cancers. Therefore, we speculated that several genes from the 24-gene signature might generate unexpected effect on the invasiveness level of these tumor types, thus leading to the opposite prognostic results. For example, INHBA (inhibin βA), a subunit of a ligand of the transforming growth factor-β superfamily, has been reported to be a tumor suppressor in DLBC [58], but a promotor in BRCA [59]. Meanwhile, TIMP3, which encodes a metalloproteinases inhibitor, also suppresses the invasion and migration of a few cancer types such as LIHC and THCA [60, 61]. In addition, Lai et al. indicated that SULF1 normally functions as a negative regulator in HNSC and loss of it potentiates growth factor signaling, enhances motility, invasiveness and inhibits stress-induced apoptosis [62]. However, considering our results from TCGA and external validation cohorts, despite these exceptions, we still firmly believe the robustness and representativeness of our invasiveness score in most cancer types.

Another important finding of this analysis was the significant positive correlation between the invasiveness score and a series of immune-associated biomarkers, as well as the higher enrichment level of several immune-checkpoint molecules in the high-invasiveness score group across multiple cancer types, implicating potential application of the score in the identification of tumor patients who are more likely to benefit from immunotherapy. This phenomenon of increased immune infiltration in higher invasiveness tumors could be partly explained by the existence of cancer-associated fibroblasts (CAFs), which are important during tumor growth, invasion, and dissemination through a paracrine fashion [63]. Evidence continues to mount that activated CAFs contribute to not only the maintenance of an inflammatory phenotype within the tumor microenvironment by the secretion of several chemokines involved in the recruitment of innate and adaptive immune cells, such as monocytes, mast cells, and T cells, but also through their acquisition of an immune-editing and immunosuppressive phenotype [63, 64]. We also found a strong correlation between the invasiveness score and the level of hypoxia, which might be due to poor perfusion and excessive oxygen consumption in advanced stage tumors. It has been reported that hypoxia-inducible transcription factors (HIFs) also participates in compromising the cytotoxic functions of immune cells that infiltrate tumors, further enhancing the malignant phenotype [65]. Therefore, in cancer patients with high-invasiveness scores, the combined use of anti-CAF therapy, anti-hypoxia therapy, and immunotherapy aimed at targeting immune checkpoint blockade might reverse the immunosuppressive tumor microenvironment, resulting in a more durable therapeutic response [66] and changing the face of anti-cancer treatment.

A number of potential therapeutic targets were identified through our correlation analysis of common DEGs in multiple cancer types and drug-response data, such as the GSK3-inhibitor SB216763, which has been reported to inhibit the proliferation of several cancers [67]. Another “core” drug, the DNA-PK inhibitor NU7441, has been demonstrated to increase cancer cell sensitivity to chemotherapy and radiotherapy [68]. Although several previous studies have shown the importance of DNA methylation, miRNA regulation, somatic mutations, and CNVs in cancer invasiveness and metastasis [46, 69], alterations in mutations and copy number seem to be mostly determined by tumor type. By contrast, our correlation analyses illustrated the complicated intersection among common invasiveness-related dysregulated genes, miRNAs targeting the 3′-UTR regions, and promoter methylation in different cancer types; we also integrated these invasiveness-associated genomic alterations and drug sensitivity in ESCA samples. Further in vitro and in vivo validations are warranted to determine the clinical relevance of these drugs in patients with different invasiveness statuses and corresponding genomic alterations.

Interestingly, we found a strong negative correlation between the invasiveness score and several metabolites, including β-alanine and isovalerylcarnitine. Vaughan et al. demonstrated β-alanine functions as an intracellular buffer in the regulation of cancer cell energetics that elicits several anti-tumor effects by suppressing glycolytic and oxidative metabolism, resulting in reduction of the total metabolic rate [70]. However, no research regarding the impact of isovalerylcarnitine and cancer has been published. A pan-cancer study focusing on the potential clinical implications of the combination of invasiveness score and metabolites will be necessary to validate our findings.

Our study still has several limitations. First, considering the spatial heterogeneity in one tumor sample, the lack of multi-loci sampling RNA-sequence data within a single tumor in these public large-scale datasets such as TCGA and GEO might weaken the predictive value of the invasiveness score. With advancements in single-cell sequence techniques, we believe this will be readily addressed in future studies. Second, the information regarding the immunotherapy process and outcome were not provided in TCGA database, thus limiting us from validating our findings on the associations between cancer immunity and invasiveness in patients receiving immunotherapy. Third, as a pan-cancer analysis, our research is aimed at identifying the common molecular alterations related to tumor invasiveness across most tumor types. However, during this process, some distinct but important features in specific cancer, such as the diverse prognostic value of one gene in different tumor types mentioned above, might be inevitably neglected. Further research beyond these common features is warranted to precisely elucidate the specific meaning of our gene signature, invasiveness score, and all of the molecular alterations, in each cancer type.

## Conclusion

In summary, by integrating multi-omics data, our large-cohort pan-cancer study provides a comprehensive atlas of genomic factors associated with tumor invasiveness and extracts common molecular alterations across tumor types, shedding light on the complex regulatory network of tumor invasiveness and may guide more precise and personalized therapeutic strategies for tumor patients.

## Supplementary information

**Additional file 1: Table S1.** The major results, as well as corresponding data sources and analytical methods in each subsection. **Table S2.** Heuristic choices used for the criteria for common molecular alteration in different datatypes.

**Additional file 2: Figure S1.** Overview of the study design. Key steps were highlighted with bold font.

**Additional file 3: Figure S4.** (**A**) Kaplan–Meier curves show the prognostic value of the invasiveness score in 27 cancer types from TCGA (except for LUAD, LUSC, and ESCA).

**Additional file 4: Figure S5.** (**A**–**F**) Independent external validation. Heatmaps exhibit the distribution of expression level of the 24 invasiveness-signature-genes in patients from high- and low-invasiveness score group, while Kaplan–Meier curves show the prognostic value of the invasiveness score in LUAD (**A**), LUSC (**B**), ESCA (**C**) patients from GEO, LUAD (**D**), LUSC (**E**) patients from our institution, and pan-cancer (**F**) patients from PRECOG.

**Additional file 5: Figure S6.** Bar plot exhibited the distribution of differentially methylated CpGs across gene regions (TSS1500, TSS200, 5′-UTR, 1st exons, 3′-UTR, body, IGR) in different cancer types.

**Additional file 6: Figure S7.** The intersection of invasiveness-related mRNAs, DNA methylation, miRNAs and response in ESCA. Downregulated genes (blue dots) in high-invasiveness group were all hyper-methylated and regulated by multiple upregulated miRNAs (green dots). The expression levels of these genes significantly correlated with the IC50 to various kinds of anti-cancer drug (red dots, |r| > 0.3, p < 0.05), while that of miRNAs displayed opposite correlation with the same drug. The pink lines represent the targeting relationship between miRNA and genes, or the correlation between miRNA/gene and drug response.

**Additional file 7: Figure S2.** (**A**) The prognostic value of the GSVA score based on other three invasiveness gene signature in LUAD patients. (**B**) Heatmaps exhibit the distribution of expression level of the 24 invasiveness-signature-genes in patients from high- and low-invasiveness score group in 30 cancer types. **Figure S3.** (**A**) The distribution of tumor stage in high- and low-invasiveness score group in different cancer types. Only cancers with reliable and available stage information were displayed here. (**B**) The circos plot exhibits invasiveness-related metabolites in BRCA patients. The length of each arc indicates the Spearman correlation between the invasiveness score and a series of metabolites. Two positively-correlated metabolites with the highest r were displayed in the plot. **Data S1.** Gene signatures mentioned in the study for GSVA/ssGSEA analysis. **Data S2.** Invasiveness score of the 9672 patients from 33 cancer types in TCGA. **Data S3.** Invasiveness-associated molecular features across 30 cancer types (high-invasiveness vs. low-invasiveness). **Data S4.** The spearman correlation of imputed drug response (GDSC) and invasiveness-related gene expression level. **Data S5.** Enriched invasiveness-related GO biological process across 30 cancer types. **Data S6.** Invasiveness related differentially expressed and methylated genes across 30 cancer types. **Data S7.** The targeting relationship and fold change (high-invasiveness vs. low-invasiveness) of invasiveness-related miRNA and genes across 30 cancer types. **Data S8.** Correlation between drug response data and the expression level of gene or miRNA and the targeting relationship between miRNA and genes in ESCA (up-regulated genes). **Data S9.** Correlation between drug response data and the expression level of gene or miRNA and the targeting relationship between miRNA and genes in ESCA (down-regulated genes).

## Data Availability

All data generated or analyzed during this study are included in this published article and its Additional files. Source data were obtained from TCGA database.
