# Supplementary material for: Multi-omics characterization and validation of invasiveness-related molecular features across multiple cancer types
Source: J Transl Med. 2021 Mar 25;19:124. doi: 10.1186/s12967-021-02773-x (PMC7995758; doi:10.1186/s12967-021-02773-x)
Supplement: Supplementary file 1 — Additional file 1: Table S1. The major results, as well as corresponding data sources and analytical methods in each subsection. Table S2. Heuristic choices used for the criteria for common molecular alteration in different datatypes. [file 12967_2021_2773_MOESM1_ESM.docx]

**Table S1.** The major results, as well as corresponding data sources and analytical methods in each subsection.

| Subsection | Data source | Analytical method | Key identification |
| --- | --- | --- | --- |
| Invasiveness score calculation | mRNA sequencing data of 30 cancer types from TCGA database | Gene set enrichment analysis (GSVA) | Based on the 24-gene signature, we found the distinct distribution patterns of invasiveness score in different cancer types. |
| Invasiveness group classification | Recurrence free survival (RFS) data from TCGA | Log-rank survival analysis | High-invasiveness scores were consistently associated with worse prognosis in most of the 30 cancer types. |
| Validation of the prognostic value of invasiveness group | ※RFS data from GEO:  LUAD: GSE30219;  LUSC: GSE29013;  ESCA: GSE53625.  ※RFS data from Zhongshan Hospital:  LUAD/LUSC.  ※ Overall survival (OS) data from PRECOG:  BLCA: GSE5479;  BRCA: GSE19783;  COAD: GSE17536;  STAD: GSE4007;  Kidney cancer: Zhao_RCC;  MESO: Gordon_Mesothelioma;  OV: GSE13876;  PAAD: GSE21501;  PRAD: GSE10645;  LUAD: ca00182. | GSVA and Log-rank survival analysis | High-invasiveness scores were consistently associated with worse prognosis in all the validation cohorts. |
| The representativeness of invasiveness score at a post-transcriptional level | Mass proteomic spectrum data of BRCA and OV from the CTPAC database | Gene set enrichment analysis (GSEA) | The 24-gene signature, which corresponds to 24 proteins, was significantly enriched in the high-invasiveness score group for both cancer types |
| Multidimensional comparison of molecular features between the high- and low-invasiveness groups | mRNA-sequencing (58387 genes), mature miRNA (approximately 2000 miRNAs), protein expression (223 proteins), DNA methylation (396,066 probes covering > 20,000 genes), somatic mutation, and CNV data from TCGA. | ﻿Fit linear model by *limma* package and adjust the p-value as false discovery rate (FDR). | Significantly different molecular features were identified. |
| Correlations between metabolites and invasiveness score | Metabolites data from the study of Tang et al. | Correlation analysis | Several metabolites, such as β-alanine (r = −0.825) and isovalerylcarnitine (r = −0.808, were significantly correlated with the score. |
| Therapeutic potential of common DEGs | Drug sensitivity and expression data from GDSC | Correlation analysis | Correlations among the anti-cancer drugs like NU7441 or KU55933 and 104 invasiveness-related DEGs such as COL3A1, A2M, and ACTN1. |
| Common invasiveness related biological processes | MSigDB database (Gene Ontology) | Functional enrichment analysis | Several key biological processes, including EMT transition, response to hypoxia, and immune activation, were enriched across multiple tumor types. |
| Differentially methylated and expressed genes (DMEGs) analysis | mRNA-sequencing and DNA methylation data from TCGA. | ﻿Fit linear model by *limma* package and adjust the p-value as FDR. | DMEGs exhibited few generalities across tumor types. |
| Intersection between invasiveness-related DEGs and differentially expressed miRNAs | mRNA-sequencing and mature miRNA data from TCGA;  miRNA-gene targeting relationship predicted by miRWalk. | Fit linear model by *limma* package and adjust the p-value as FDR. | 21 miRNAs significantly overexpressed in the high-invasiveness group and their potential target genes were identified. |
| Integrative analysis in ESCA | mRNA-sequencing, methylation, and mature miRNA data of ESCA from TCGA;  miRNA-gene targeting relationship predicted by miRWalk. | Fit linear model (FDR) and correlation analysis. | Gene-methylation-miRNA-drug regulatory network. |

**Table S2.** Heuristic choices used for the criteria for common molecular alteration in different datatypes.

| Analysis | Data type | Criteria |
| --- | --- | --- |
| Differentially expressed genes (DEGs) | RNA sequencing data from TCGA | Genes significantly differentially expressed in at least **ten** cancer types. |
| Functional enrichment analysis | MSigDB database (Gene Ontology) | Pathways that were significantly enriched in at least **ten** cancer types |
| Differentially methylated and expressed genes (DMEGs) | RNA sequencing and methylation data from TCGA | Genes significantly differentially methylated and expressed in at least **three** cancer types |
| Differentially expressed miRNAs | miRNA data from TCGA | miRNAs significantly differentially expressed in at least **16** cancer types |
| Common miRNA-target genes | DEGs | DEGs regulated by at least 5 miRNAs mentioned above |
