# Supplementary figures and images for: Multi-omics characterization and validation of invasiveness-related molecular features across multiple cancer types
Source: J Transl Med. 2021 Mar 25;19:124. doi: 10.1186/s12967-021-02773-x (PMC7995758; doi:10.1186/s12967-021-02773-x)

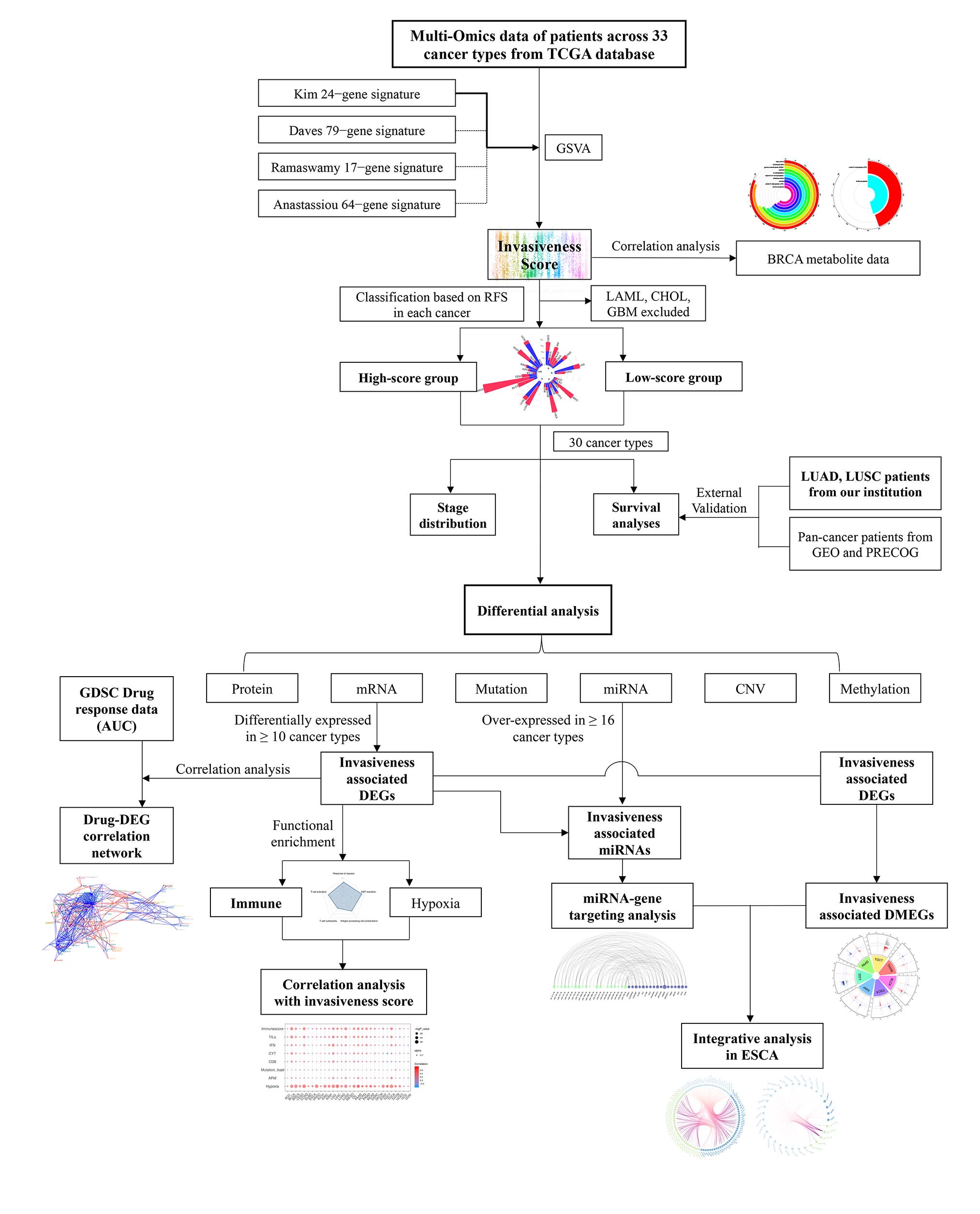

Supplement: Supplementary file 2 — Additional file 2: Figure S1. Overview of the study design. Key steps were highlighted with bold font. [file 12967_2021_2773_MOESM2_ESM.jpg]

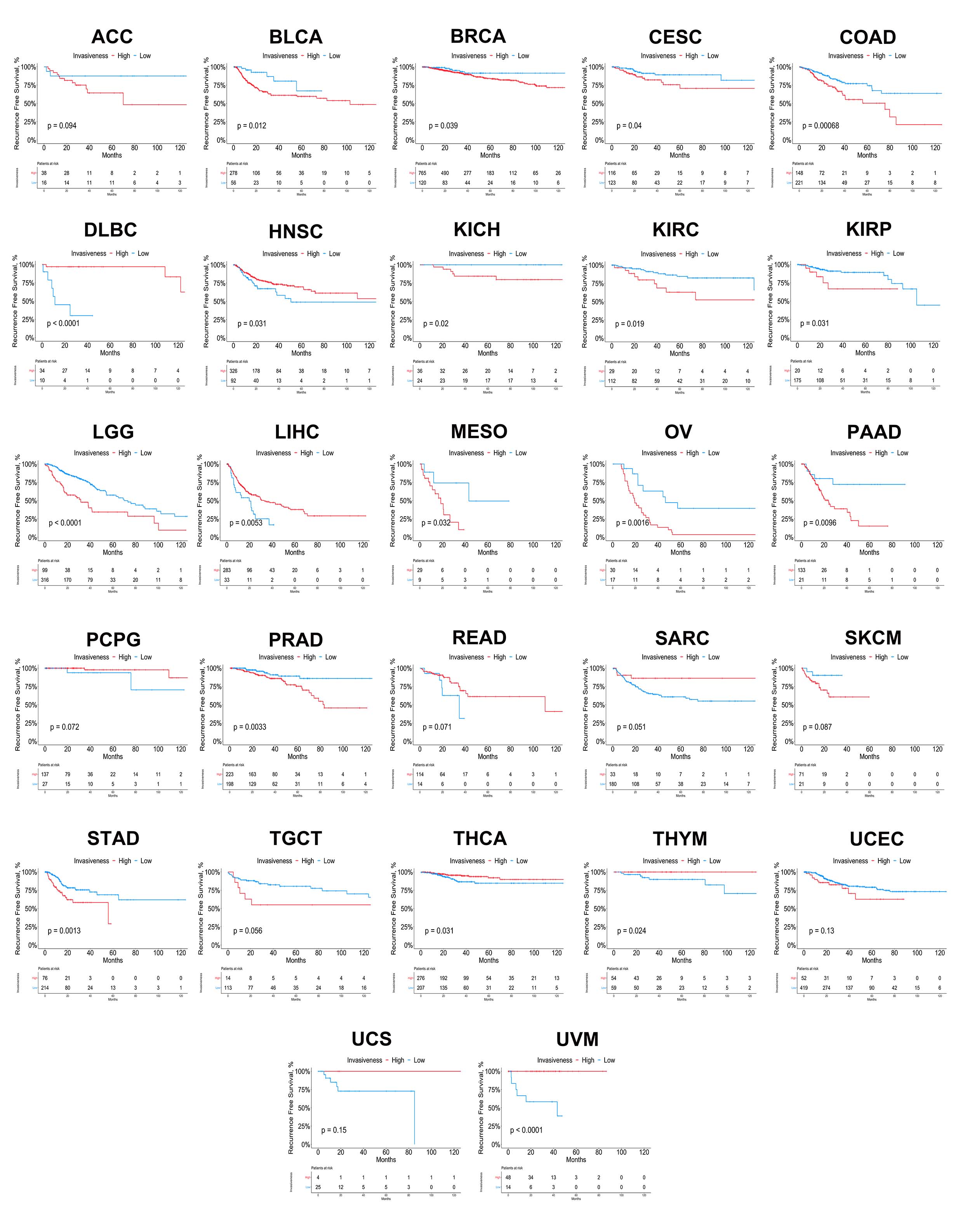

Supplement: Supplementary file 3 — Additional file 3: Figure S4. (A) Kaplan–Meier curves show the prognostic value of the invasiveness score in 27 cancer types from TCGA (except for LUAD, LUSC, and ESCA). [file 12967_2021_2773_MOESM3_ESM.jpg]

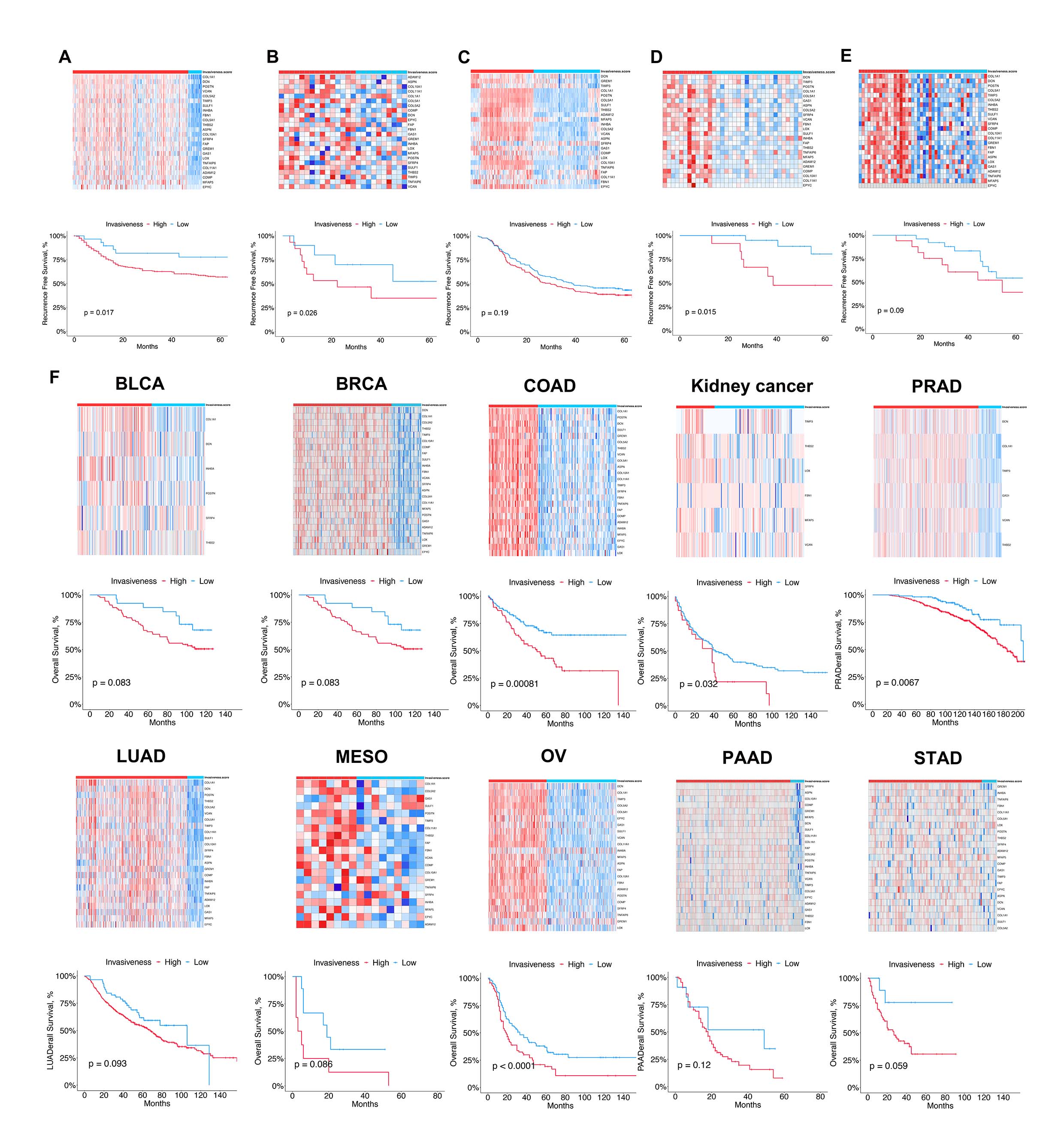

Supplement: Supplementary file 4 — Additional file 4: Figure S5. (A–F) Independent external validation. Heatmaps exhibit the distribution of expression level of the 24 invasiveness-signature-genes in patients from high- and low-invasiveness score group, while Kaplan–Meier curves show the prognostic value of the invasiveness score in LUAD (A), LUSC (B), ESCA (C) patients from GEO, LUAD (D), LUSC (E) patients from our institution, and pan-cancer (F) patients from PRECOG. [file 12967_2021_2773_MOESM4_ESM.jpg]

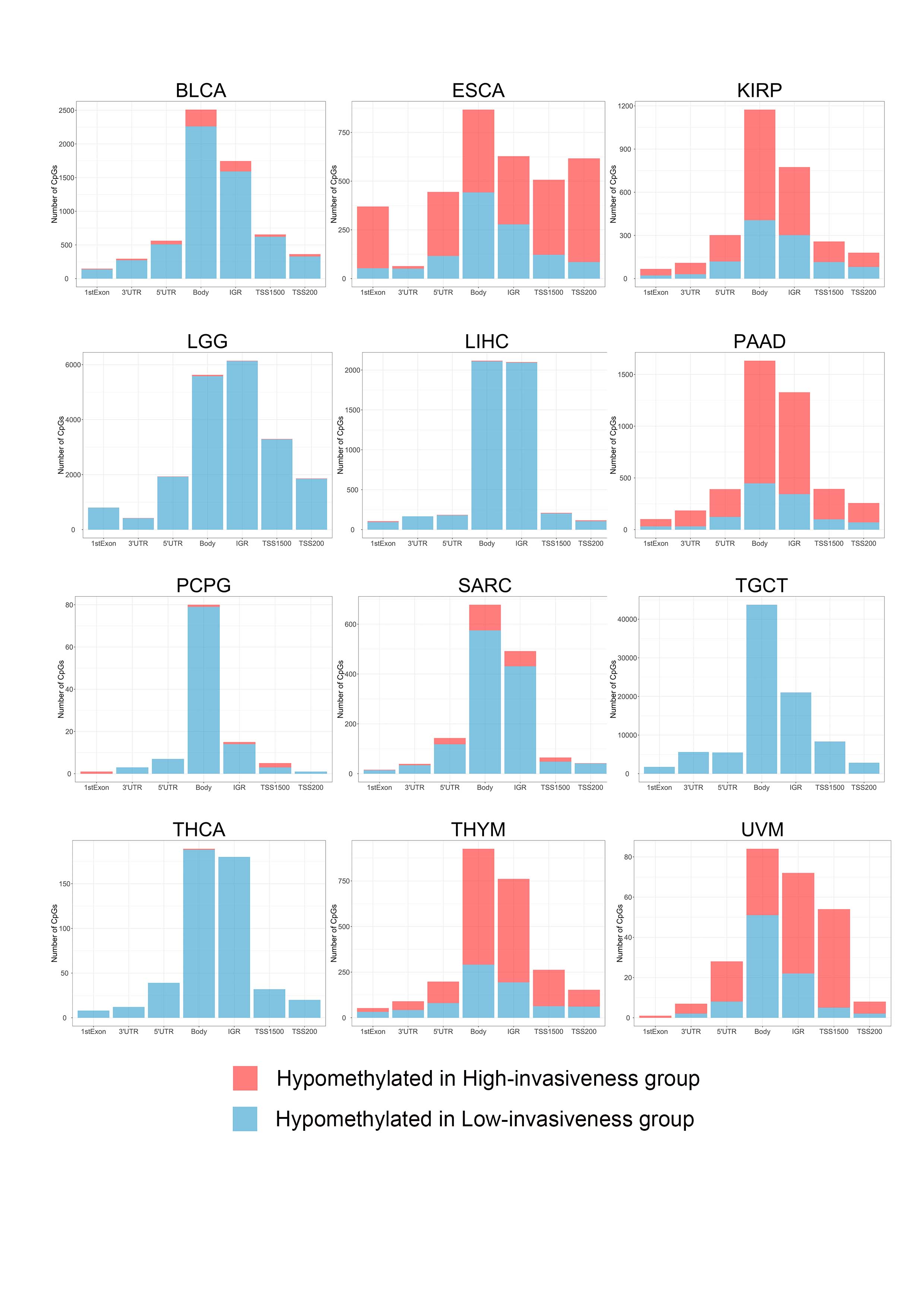

Supplement: Supplementary file 5 — Additional file 5: Figure S6. Bar plot exhibited the distribution of differentially methylated CpGs across gene regions (TSS1500, TSS200, 5′-UTR, 1st exons, 3′-UTR, body, IGR) in different cancer types. [file 12967_2021_2773_MOESM5_ESM.jpg]

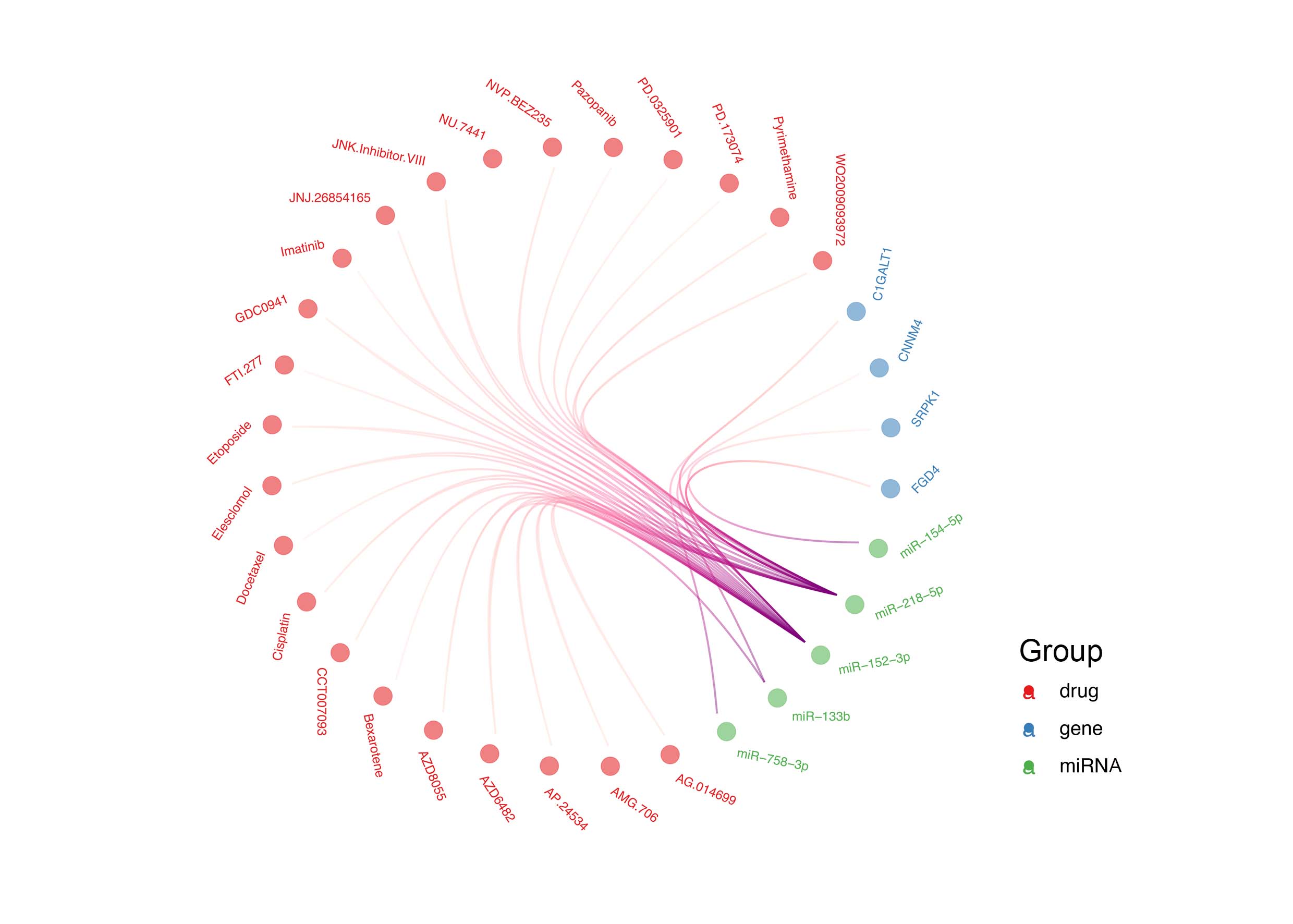

Supplement: Supplementary file 6 — Additional file 6: Figure S7. The intersection of invasiveness-related mRNAs, DNA methylation, miRNAs and response in ESCA. Downregulated genes (blue dots) in high-invasiveness group were all hyper-methylated and regulated by multiple upregulated miRNAs (green dots). The expression levels of these genes significantly correlated with the IC50 to various kinds of anti-cancer drug (red dots, |r| > 0.3, p < 0.05), while that of miRNAs displayed opposite correlation with the same drug. The pink lines represent the targeting relationship between miRNA and genes, or the correlation between miRNA/gene and drug response. [file 12967_2021_2773_MOESM6_ESM.jpg]
